# Comparison of the PUSH Band 2.0 and Vicon Motion Capture to Measure Concentric Movement Velocity during the Barbell Back Squat and Bench Press

**DOI:** 10.3390/sports11010006

**Published:** 2022-12-28

**Authors:** Edward Z. Pelka, Carter Gadola, Daniel McLaughlin, Eric Slattery, Randal P. Claytor

**Affiliations:** 1 Department of Kinesiology, Nutrition and Health, Miami University, Oxford, OH 45056, USA; 2 Exercise Science and Exercise Physiology Program, Kent State University, Kent, OH 44242, USA

**Keywords:** wearable technology, velocity-based training, resistance exercise

## Abstract

The purpose of this investigation was to compare concentric movement velocity (CMV) measured with the PUSH Band (v2.0) and a Vicon motion capture system (MC) during the back squat (SQ) and the bench press (BP) resistance exercises (RE). Twelve resistance-trained males (26.0 ± 5.5 years; 175.6 ± 4.9 cm; 96.3 ± 15.8 kg) completed ten repetitions at 50% of one-repetition maximum (1RM), and six repetitions at 75% 1RM for both BP and SQ. Four PUSH devices were utilized and attached to the subject’s right forearm, the center barbell, left and right sides of the barbell. MC markers were placed on top of each PUSH device. An overall analysis using a series of least-squares means contrasts suggested CMV did not differ (*p* > 0.05) between measurement technologies when position, RE, intensity and repetitions were combined. PUSH exhibited the highest Intraclass Correlation Coefficients (ICC = 0.835–0.961) and Pearson Product-Moment Correlation Coefficients (*r* = 0.742–0.949) at the arm and center barbell locations when compared with MC. The measurement of CMV between MC and PUSH compares favorably during moderate (i.e., 50%) and high (75%) intensity SQ and BP RE. These data indicate individuals can use the PUSH band v2.0 to accurately monitor CMV within a RE set for SQ and BP RE.

## 1. Introduction

Velocity-based RE training (VBT) has become a popular approach for strength and conditioning personnel to enhance sport performance. Strength and conditioning personnel can utilize VBT to improve motivation and performance throughout training [1]. The purpose of VBT is to monitor CMV in real time [2], as acute muscular fatigue has been reported to result in decreases in CMV [3]. By using monitoring technology, strength and conditioning personnel are able to detect these changes in CMV and adjust training loads in real time. It has been suggested VBT should utilize velocity thresholds of 10–20% to optimize performance adaptations [1]. Despite performing less work, recent research suggests VBT may provide a better means of improving muscular strength and athletic performance [4,5] than more traditional methods of RE training. Additionally, monitoring CMV at multiple training loads enables strength and conditioning personnel to not only predict an athlete’s 1RM [6], but also determine how many more repetitions an athlete can perform in a set until failure [7].

Currently, an issue with VBT is establishing valid and reliable assessment technologies for measuring CMV that is inexpensive and easy for strength and conditioning personnel to use in a group setting; linear transducers (LT) and MC are cumbersome and expensive but have been reported to provide accurate measures of CMV. Thus, LT and MC devices may be impractical for everyday use during training. Additionally, there is a paucity of investigations comparing cheap commercially available wearable technologies with gold standard devices to measure CMV during RE training.

PUSH Band v2.0 (PUSH) (PUSH Inc, Toronto, ON, Canada) is a six-axis inertial sensor that collects data at 1000 Hz via its 3D accelerometer and gyroscope. PUSH provides in real time CMV feedback on a rep-by-rep basis by connecting to an app on smartphones or tablets. Several published studies assessed PUSH during the SQ [8,9,10,11,12,13] and the BP [10,12,14,15,16]. However, there is a discrepancy in the literature regarding the ability of the PUSH Band to accurately measure CMV. Despite these discrepancies, a recent systematic review suggests PUSH is valid and reliable at measuring CMV regardless of the exercise movement performed [17].

In reference to this study, several published studies have compared PUSH to Vicon MC during the SQ [10,11,13], and the BP [10,14]. Additionally, no studies, to our knowledge, have compared measurements of CMV with PUSH versus Vicon MC across RE sets of six to ten repetitions during SQ and BP. The primary purpose of this investigation was to compare average CMV between PUSH and MC during SQ and BP completed at 50% 1RM and 75% 1RM. A secondary purpose was to determine which PUSH placement site provided the most accurate measures of CMV as compared to MC. The final purpose was to measure CMV across multiple repetitions completed during a single set of SQ and BP RE. We hypothesized that PUSH would provide accurate and reliable CMV measurements, and the arm and center barbell placements would provide the most accurate measurements of the four placement locations.

## 2. Materials and Methods

### 2.1. Experimental Approach to the Problem

Participants reported to the laboratory once to complete ten repetitions at 50% 1RM, and six repetitions at 75% 1RM for both BP and SQ. The order of the RE mode was counterbalanced. Four PUSH devices were used and attached to the subject’s right arm, the left side of the barbell (LB), the right side of the barbell (RB) and the center of the barbell (CB), with MC markers placed directly on top of each PUSH. This design allowed us to compare measures of CMV between PUSH and MC during multiple resistance exercises, intensities, repetitions and to determine which measurement location provided the most accurate measurement of CMV when comparing PUSH with MC.

### 2.2. Subjects

Twelve resistance trained males from DI collegiate football and collegiate club power-lifting teams volunteered to participate in this study. Any individuals who reported any past (six months) or current injury were excluded from this study. Anthropometric measurements were taken, and body composition was assessed with a bioelectrical impedance device (Inbody 770—Inbody USA, Cerritos, CA, USA) (Table 1). All subjects were informed of the study design as well as risks and benefits of participation, prior to the start of data collection. This study protocol was in accordance with ethical requirements and approved by the University’s Institutional Review Board.

### 2.3. Procedures

Prior to the experimental protocol, subjects completed a standardized warm-up of cycling on a stationary ergometer for five minutes, followed by a personalized warm-up. Subjects were randomly assigned to perform BP or SQ, first. Each condition was performed in a 2D Smith Machine (Jones Commercial—BODYCRAFT, Lewis Center, OH, USA), which moves horizontally and vertically. PUSH devices were attached to the medial aspect of the LB and RB sleeves, and CB, in bar mode, and the right arm in body mode, as recommended by the manufacturer. Each PUSH was labeled (i.e., LB, RB, CB, and ARM) and used at the same location and connected to the same Apple Ipad via Bluetooth for each participant. Four separate Ipads were used, one for each PUSH band. MC markers were placed directly on top of each PUSH. Prior to data collect, a ten-camera digital MC (Vicon Motion Systems, Oxford, UK) system was calibrated to 3000 frames for each session. Each subject completed ten repetitions at 50% and six repetitions at 75% of self-reported 1RM, with five min rest between each set. The rational for using self-report 1RM is as follows; all subjects in our study were from DI collegiate football and collegiate club power-lifting teams. These individuals consistently trained at percentages of known 1RM values. Subjects were instructed to perform the concentric portion of each repetition at maximal effort and velocity, and perform the eccentric portion of each repetition in a slow and controlled manner. After the RE trails, subjects completed a form detailing the comfort of each exercise in the 2D Smith Machine. The 5-point scale range from; 1 (extremely uncomfortable—inhibited me greatly), 2 (very uncomfortable—inhibited me somewhat), 3 (a little uncomfortable—inhibited me a little), 4 (comfortable—did not inhibit me), 5 (completely comfortable—similar to free weights). Subjects ranked the SQ on average at 3.8 and the BP at 3.7.

### 2.4. Statistical Analyses

PUSH data was recorded in real-time and stored on separate Ipads for each device location. MC marker data for displacement and velocity was recorded and stored on Vicon Nexus software (Vicon Motion Systems, Oxford, UK) at 100 Hz, and exported to Microsoft Excel for analysis. Average CMV was calculated for each repetition during the concentric phase, which was used for analysis and was defined as the time difference between the 1st positive and 1st negative vertical velocity. Linear Mixed Model, with least-squares means post hoc analyses were performed to compare PUSH and MC CMV, with α = 0.05. Furthermore, ICC, Pearson’s product-moment correlation coefficients (r), MinMax Accuracy, Mean Absolute Error (MAE), and Mean Error (ME) were used to compare the viability of PUSH versus MC CMV. Additionally, Bland–Altman plots and then least products regression analyses were used to determine indices of bias. The lmodel2 package in R-statistical programming [18] was used for performing least products regression analysis to compare the CMV between MC and PUSH ([MC*PUSH]/2) to the difference in CMV between MC and PUSH (MC—PUSH) for each intensity, exercise, device placement, and repetition.

## 3. Results

**Figure 1:**
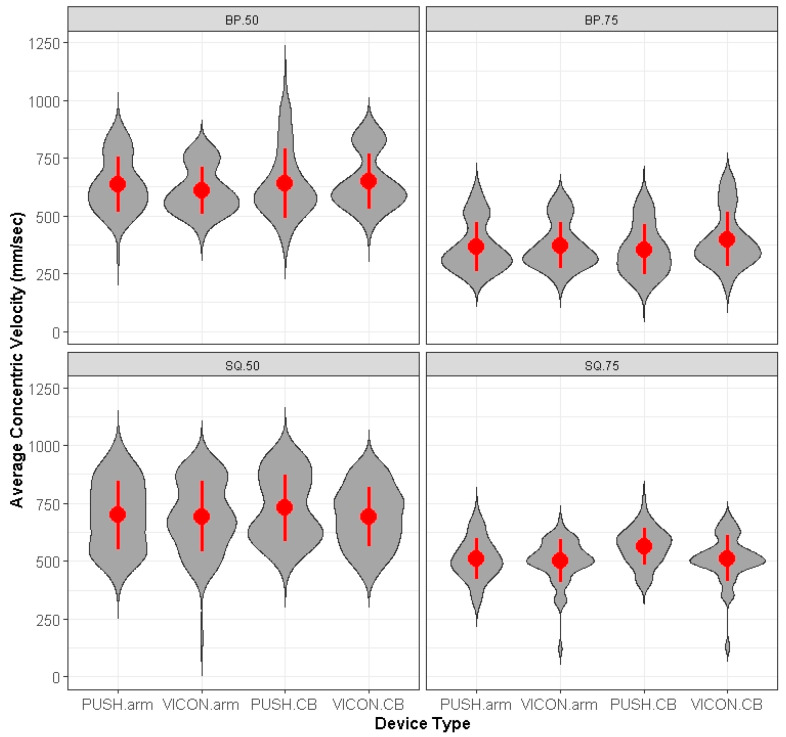
Represents violin plots of PUSH versus MC mean and standard deviation CMV measurements at specific intensities, RE and placement sites. CB = center barbell; BP = bench press; SQ = squat; 50 = 50% intensity; 75 = 75% intensity.

Out of 1544 data points for each device, there were 35 (2.3%) missed measurements with the VICON and 57 (3.7%) missed measurements for the PUSH. When one device missed a measurement at a location, the data was subsequently removed at that same location, exercise, intensity, and repetition for the other device. In all 92 measurements were removed from analysis. Thus, from 1544 measurements, we analyzed and compared 1452 measurements between each device. All missed measurements for PUSH occurred on the right and left barbell placement locations at 50% intensity. An overall analysis using a series of least-squares means, post hoc contrasts showed no significant differences (*p* > 0.05) between PUSH and MC CMV, when position, RE, intensity, and repetition were incorporated into the Linear Mixed Model. Figure 1 represents PUSH and VICON mean CMV and CMV dispersion for each RE intensity and exercise.

In Table 2, the MAE, a measure of average error for each set between PUSH and MC ranged from 0.04 m/s to 0.11 m/s. Accuracy for PUSH ranged from 86.1% to 93.9%. Accuracy was determined by using function searches for the minimum value between PUSH and MC for each person and then divided by the maximum value between PUSH and MC. The mean was then used between devices for min/max ratios to calculate accuracy. A two-way random effects model was chosen for ICC and ranged from 0.671 to 0.961. Very strong correlations (r) were present and ranged from 0.722 to 0.949. ME, a measure of the direction of error, ranged from—0.03 to 0.06. Table 2 suggests high reliability between the SQ and BP at the arm and CB locations (ICC = 0.835–0.961); however, these values decreased at the RB and LB locations (ICC = 0.671–0.910).

In Table 3, ICC, MAE and accuracy are shown when comparing the first half versus the second half of the repetitions in each set. The 50% intensity includes five repetitions in the first half and five repetitions in the second half, while the 75% intensity includes three repetitions in the first half and three repetitions in the second half. This data suggests PUSH accuracy remains constant over a set of six to ten repetitions when compared with MC.

**Figure 2:**
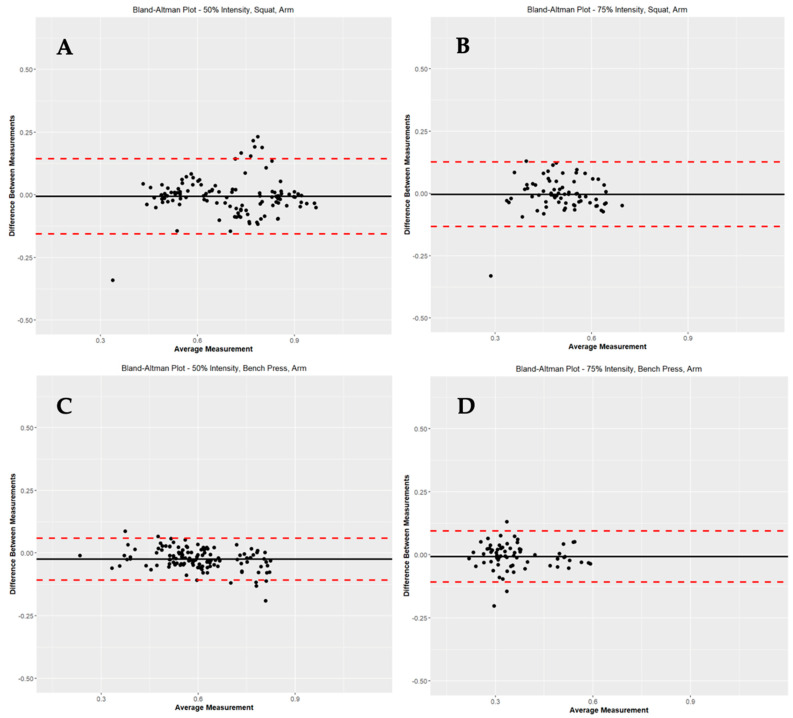
Represents Bland–Altman Plots of PUSH versus MC for the arm position at 50% squat (**A**), 75% squat (**B**), 50% bench press (**C**) and 75% bench press (**D**).

In Table 4, comparisons were made between CMV at the four locations. When comparing MC to MC, there were significant differences in CMV based on site locations (MC arm to MC CB, *p* < 0.05; MC arm to MC LB, *p* < 0.05; MC arm to MC RB, *p* < 0.05; MC CB to MC RB, *p* < 0.05). However, when PUSH was compared to MC at the same location (I.e., PUSH arm to MC arm, PUSH CB to MC CB, PUSH LB to MC LB, PUSH RB to MC LB), there were no significant differences in CMV. Figure 2 represents Bland-Altman Plots of PUSH versus MC at 50% and 75% SQ and BP for the Arm location. 

Descriptive statistics from MC and PUSH CMV, mean differences and 95% Confidence Intervals (CI) around the mean differences are presented in Table 5. These data suggest PUSH CMV is not significantly higher or lower than MC across intensity, condition or device position. The data were then analyzed using least products regression analysis to evaluate fixed and/or proportional bias when comparing PUSH with MC CMV. Results of the least products regression analyses are presented in Table 6, which suggests PUSH has significant fixed and proportional bias for CMV regardless of intensity and device position. Fixed bias indicates PUSH CMV differs from MC CMV by a constant amount and proportional bias indicates PUSH CMV differs from MC CMV by an amount proportional to MC CMV.

## 4. Discussion

To our knowledge, this is the first investigation to compare PUSH to MC during the SQ and BP RE at training intensities and volumes that most mimic actual training loads. PUSH provided accurate measures of CMV during the SQ and BP RE, when compared to MC (See Table 2 and Table 3). Table 2 indicates PUSH and MC CMV are similar for each condition. Overall, PUSH arm resulted in the best measures of CMV, closely followed by the CB; these positions exhibited highest measures of ICCs, least MAE and indicators of bias (see Table 2 and Table 3). However, there are some discrepancies in the literature regarding the comparability of PUSH CMV with criterion measurement systems [8,9,11,12,13,14,15,16,19,20].

Discrepancies between our data and other published studies regarding PUSH accuracy are likely due to differences in measurement methodologies used to monitor CMV. We used a ten-camera MC system with optical markers placed directly on top each PUSH. CMV was compared between PUSH and MC at various intensities and rep ranges for each placement site. Previous studies using LT as the gold-standard measurement technology to compare PUSH with could not make the same comparisons [9,12,16,19]. As a result, these data lead researchers to conclude PUSH results in poor validity and/or reliability during RE across various intensities. The differences between these previous investigations and our data are likely the result of different measurement methodologies. Several studies placed the LT on the outside of the barbell and PUSH on the subject’s forearm [9,12,16,19], while others placed PUSH on the subject’s forearm, with a single MC marker on the outside of the barbell [11,13,15]. When comparing MC to MC, our data suggests there are significant differences in CMV at different barbell locations (MC arm to MC CB, *p* < 0.05; MC arm to MC LB, *p* < 0.05; MC arm to MC RB, *p* < 0.05; MC CB to MC RB, *p* < 0.05). However, when PUSH was compared to MC at the same location (I.e., PUSH arm to MC arm, PUSH CB to MC CB, PUSH LB to MC LB, PUSH RB to MC LB), there were no significant differences in CMV (Table 4). Our data suggests when PUSH was placed on the subject’s arm and the criterion measurement system (MC) was placed at a different location on the barbell, differences in CMV were recorded between measurement techniques. The results from our study can potentially explain why studies that compared PUSH CMV at the arm location to a criterion measurement system placed on one end of the barbell reported poor CMV validity and/or reliability; CMV was significantly different at different barbell locations when measured either by PUSH or MC. This is likely a function of differences in individual bar paths. During RE the bar may move away from a perfect parallel position, resulting in differences in peak and average CMV near the ends of the barbell, regardless of measurement methodologies. A recent investigation reported similar results to ours in which differences were observed in CMV based on different device placement locations [11]. Therefore, device placement must be taken into consideration when performing CMV validity and/or reliability measures.

When previous studies utilized a similar measurement methodology, placing a MC marker directly on top of PUSH [14,20] or placing PUSH on the arm and MC markers on the elbow and hand [21], the data suggested PUSH provides reliable measures of CMV during a barbell bench press [14], a counter movement jump [20], a biceps curl and shoulder press RE [21]. The results reported in these studies are in general agreement with the results of our study. This suggests PUSH accurately monitors CMV when compared to MC (ICC = 0.835–0.961; *r* = 0.742–0.949) at the arm and CB locations during moderate to intense SQ and BP RE. Similarities in our results and these previous studies [14,20,21] may be explained by the similarities in the measurement methodologies that were employed; we compared PUSH CMV with MC CMV by placing MC markers on PUSH devices rather than measuring PUSH at the arm position and then comparing this value with the criterion CMV values measured at different barbell locations (i.e., end of right or left barbell) [9,12,13,15,16,19].

One unique purpose of our study was to determine if and/or to what degree PUSH measures of CMV were in agreement with MC in the ability to track changes in CMV (i.e., loss in CMV) across repetitions completed within a set of RE. To our knowledge, no other published studies have compared PUSH CMV with MC CMV across multiple repetitions, while using intensities that that mimic actual training loads (i.e., 75% 1RM), and while employing RE that are typically used for training purposes. Past studies [8,9,12,14,15,16,19], all used a similar methodology of performing one to three repetitions at given intensities, while our study measured CMV for six consecutive repetitions at 75% 1RM and ten consecutive repetitions at 50% 1RM for both SQ and BP RE to determine if PUSH could track velocity loss across a RE set. The purpose of VBT is to monitor fluctuations in CMV in real time [2], as acute muscular fatigue has been reported to influence CMV [3] and a drop in CMV below a predetermined threshold can be used to terminate a set [4]. The information in Table 3, suggests PUSH can accurately monitor velocity loss in real time as changes in CMV compared favorably between PUSH and MC across the first and second halves of a set, for SQ and BP at 50% and 75% 1RM. These data suggest PUSH can be used to accurately monitor CMV loss over the course of a set of both SQ and BP RE using a Jones machine. Since subjects reported an average comfort score of 3.7–3.8 (on a Likert scale of 0–5) for the Jones machine compared to the use of free-weights during the BP and SQ RE, respectively, the use of the Jones machine may have inhibited the subjects’ movement comfort when performing these exercises. Therefore, future investigations should determine whether differences arise in CMV and with the accuracy of PUSH between the use of the Jones machine and the use of free-weights during SQ and BP RE. This will provide better insight into whether strength and conditioning personnel can effectively use the PUSH in everyday strength and conditioning settings for the assessment of CMV and the use of VBT.

## 5. Conclusions

In conclusion, there were no statistically significant differences between PUSH and MC CMV as a function of RE, device placement, intensity and repetition number. On a rep-by-rep basis, PUSH arm location provided the most accurate measurement of CMV for SQ and BP RE when compared with MC, closely followed by the CB location. Overall, these data suggest PUSH provides accurate and reliable real-time measurements of CMV when compared to MC and PUSH can effectively be used to detect changes in CMV on a rep-by-rep basis to facilitate the use of VBT.

## Figures and Tables

**Table 1:** Physical characteristics.

Variable	Mean ± SD
Age (years)	26.0 ± 5.5
RE experience (years)	10.7 ± 5.6
Height (cm)	175.6 ± 4.9
Weight (kg)	96.3 ± 15.8
% fat	23.4 ± 8.4
Relative SQ 1RM	1.77 ± 0.30
Relative BP 1RM	1.23 ± 0.23

RE = resistance exercise; SQ = back squat; BP = bench press; 1RM = one-repetition maximum.

**Table 2:** Measures of accuracy and bias between PUSH and MC.

				Reliability		Error	Bias
RE	Intensity	Position	Accuracy	Correlation (r)	ICC	MAE (m/s)	ME
BP	50	Arm	93.80	0.949	0.961	0.04	0.03
SQ	50	Arm	92.93	0.870	0.931	0.05	0.01
BP	75	Arm	89.66	0.880	0.936	0.04	0.01
SQ	75	Arm	90.88	0.742	0.852	0.05	0.01
BP	50	CB	90.66	0.871	0.922	0.06	−0.02
SQ	50	CB	93.91	0.947	0.955	0.05	0.04
BP	75	CB	86.14	0.847	0.890	0.05	−0.03
SQ	75	CB	90.12	0.831	0.835	0.06	0.05
BP	50	LB	84.80	0.722	0.809	0.11	−0.03
SQ	50	LB	91.24	0.815	0.889	0.07	0.03
BP	75	LB	86.71	0.839	0.910	0.05	−0.02
SQ	75	LB	89.41	0.863	0.835	0.07	0.06
BP	50	RB	87.76	0.773	0.828	0.10	−0.01
SQ	50	RB	92.66	0.912	0.894	0.06	0.03
BP	75	RB	87.74	0.878	0.867	0.05	−0.02
SQ	75	RB	87.570	0.826	0.671	0.08	0.07

BP = bench press; SQ = squat; CB = center bar; LB = left bar; RB = right bar.

**Table 3:** Measures of accuracy for first half compared to last half of repetitions.

			First Half of Repetitions			Last Half of Repetitions		
RE	Intensity	Position	ICC	MAE (m/s)	Accuracy (%)	ICC	MAE (m/s)	Accuracy (%)
BP	50	Arm	0.952	0.04	93.63	0.969	0.04	93.98
SQ	50	Arm	0.932	0.05	93.01	0.931	0.05	92.84
BP	75	Arm	0.933	0.06	90.84	0.913	0.06	90.48
SQ	75	Arm	0.974	0.04	95.37	0.939	0.06	92.45
BP	50	CB	0.873	0.10	86.69	0.718	0.12	82.94
SQ	50	CB	0.922	0.07	92.29	0.848	0.08	90.21
BP	75	CB	0.843	0.09	88.32	0.815	0.10	87.16
SQ	75	CB	0.960	0.05	93.71	0.925	0.07	91.59
BP	50	LB	0.937	0.04	91.03	0.899	0.04	88.95
SQ	50	LB	0.854	0.05	91.08	0.842	0.05	90.14
BP	75	LB	0.803	0.08	82.82	0.941	0.04	89.33
SQ	75	LB	0.861	0.04	91.75	0.843	0.06	89.44
BP	50	RB	0.867	0.05	88.62	0.925	0.05	86.25
SQ	50	RB	0.867	0.06	90.31	0.817	0.06	89.26
BP	75	RB	0.903	0.06	86.17	0.925	0.04	88.60
SQ	75	RB	0.828	0.07	88.10	0.789	0.078	87.37

BP = bench press; SQ = squat; CB = center bar; LB = left bar; RB = right bar.

**Table 4:** Comparison of barbell concentric movement velocity at different site locations.

Contrast	Estimate	SE	Df	T-Ratio	*p*-Value
MC arm to MC CB	−0.03	0.01	2886.22	−4.39	*0.00* *
MC arm to MC LB	−0.04	0.01	2886.05	−6.10	*0.00* *
MC arm to MC RB	−0.04	0.01	2886.03	−7.83	*0.00* *
MC CB to MC LB	−0.01	0.01	2886.30	−1.63	0.73
MC CB to MC RB	−0.02	0.01	2886.19	−3.27	*0.02* *
MC LB to MC RB	−0.01	0.01	2886.11	−1.64	0.72
Push arm to MC arm	0.01	0.0	2886.00	2.14	0.39
Push CB to MC CB	0.01	0.0	2886.00	2.14	0.39
Push LB to MC LB	0.01	0.0	2886.00	2.14	0.39
Push RB to MC RB	0.01	0.0	2886.00	2.14	0.39

MC = motion capture; CB = center barbell; RB = right barbell; LB = left barbell. * Denotes significance.

**Table 5:** Mean (SD) for MC and PUSH CMV with mean difference and 95% Confidence Intervals; by intensity, exercise and device position. If the 95% Confidence Interval does not cross 0, the mean difference is considered significantly different.

	SQ 50 Arm (*n* = 115)	SQ 50 CB (*n* = 100)	BP 50 Arm (*n* = 120)	50 BP CB (*n* = 116)	SQ 75 Arm (*n* = 70)	SQ 75 CB (*n* = 59)	BP 75 Arm (*n* = 66)	BP 75 CB (*n* = 72)
MC	0.697 (0.151)	0.672 (0.133)	0.587 (0.117)	0.621 (0.142)	0.505 (0.094)	0.514 (0.102)	0.361 (0.097)	0.386 (0.117)
PUSH	0.703 (0.149)	0.707 (0.148)	0.611 (0.132)	0.603 (0.168)	0.507 (0.091)	0.559 (0.087)	0.368 (0.098)	0.346 (0.102)
Mean Diff.	−0.006	−0.035	−0.024	0.017	−0.002	−0.045	−0.007	0.040
(95% CI)	(−0.156, 0.144)	(−0.129, 0.059)	(−0.107, 0.059)	(−0.144, 0.179)	(−0.132, 0.127)	(−0.153, 0.063)	(−0.108, 0.095)	(−0.089, 0.168)

MC = motion capture; CB = center barbell; SQ = squat; BP = bench CI = Confidence Interval.

**Table 6:** Results of least product regression analyses by intensity, exercise and device position. If the 95% CI for the slope does not include 1.0, proportional bias is present (*). If the 95% CI for the intercept does not include 0, fixed bias is present (**).

	SQ 50 Arm	SQ 50 CB	BP 50 Arm	50 BP CB	SQ 75 Arm	SQ 75 CB	BP 75 Arm	BP 75 CB
Slope	1.897	−2.887	−2.889	−1.815	1.308	1.639	−1.819	1.590
(95% CI)	(1.577, 2.283) *	(−3.488, −2.390) *	(−3.423, −2.438) *	(−2.161, −1.524) *	(1.029, 1.662) *	(1.274, 2.109) *	(−2.329, −1.421) *	(1.265, 2.000) *
Intercept	0.711	0.588	0.528	0.643	0.509	0.611	0.352	0.303
(95% CI)	(0.709, 0.713) **	(0.567, 0.605) **	(0.515, 0.539) **	(0.638, 0.649) **	(0.509, 0.511) **	(0.594, 0.632) **	(0.349, 0.355) **	(0.287, 0.316) **

CB = center barbell; SQ = squat; BP = bench; CI = Confidence Interval.

## Data Availability

Not applicable.
